# Genetic analysis of enteropathogenic *Escherichia coli* (EPEC) adherence factor (EAF) plasmid reveals a new deletion within the EAF probe sequence among O119 typical EPEC strains

**DOI:** 10.1186/s12866-015-0539-9

**Published:** 2015-10-05

**Authors:** Nathalia B. Teixeira, Thais C. G. Rojas, Wanderley D. da Silveira, Cecília Matheus-Guimarães, Neusa P. Silva, Isabel C. A. Scaletsky

**Affiliations:** Departamento de Microbiologia, Imunologia e Parasitologia, Universidade Federal de São Paulo, Rua Botucatu, 862, 3 andar, São Paulo, 04023-062 São Paulo Brazil; Departamento de Genética, Evolução e Bioagentes, Instituto de Biologia, Universidade Estadual de Campinas, Campinas, Brazil; Disciplina de Reumatologia, Universidade Federal de São Paulo, São Paulo, Brazil

**Keywords:** Enteropathogenic *Escherichia coli*, EAF plasmid

## Abstract

**Background:**

Enteropathogenic Escherichia coli (EPEC) are classified into typical and atypical strains based on the presence of the E. coli adherence factor (EAF) plasmid. The EAF plasmid contains the bfp (bundle-forming pilus) operon and the perABC (plasmid encoded regulator) gene cluster. A 1-kb cryptic region of EAF plasmid has been widely used as a genetic probe for EPEC detection. However, some EPEC strains may harbor an EAF plasmid lacking the EAF probe sequence, which makes the differentiation between typical and atypical a complex task. In this study, we report the genetic analysis of the EAF plasmid-encoded genes in a collection of EPEC clinical isolates.

**Methods:**

A total of 222 EPEC clinical isolates, which were previously classified as typical (n = 70) or atypical (n = 152) by EAF probe reactivity, were screened for the presence of different EAF sequences by PCR and DNA hybridization.

**Results:**

All typical strains possessed intact *bfpA* and *perA* genes, and most of them were positive in the PCR for EAF probe sequence. However, a subset of 30 typical strains, 22 of which belonged to O119 serogroup, presented a 1652 pb deletion in the region between 1093-bp downstream *perC* and 616-bp of the EAF fragment. The *bfpA, bfpG*, and *per* genes were found in all typical strains. In addition, 32 (21 %) atypical strains presented the *perA* gene, and 20 (13.2 %) also presented the *bfpA* gene. Among the 32 strains, 16 belonged to the O119:H2, O119:HND, and ONT:HND serotypes. All 32 atypical strains contained *perA* mutation frameshifts and possessed an IS*1294* element upstream of the *per* operon as detected by PCR followed by restriction fragment length polymorphism (RFLP) typing and multiplex PCR. Among the 20 *bfpA* probe-positive strains, eight O119 strains possessed deletion in the *bfp* operon at the 3′end of *bfpA* due to an IS*66* element.

**Conclusion:**

Our data show that typical O119 strains may contain a deletion within the EAF probe sequence not previously reported. This new finding suggests that care should be taken when using the previously described EAF PCR assay in epidemiological studies for the detection of typical O119 strains. In addition, we were able to confirm that some atypical strains carry vestiges of the EAF plasmid.

## Background

Enteropathogenic *Escherichia coli* (EPEC) are an important cause of infantile diarrhea in developing countries, particularly in Brazil [1–3]. EPEC strains produce a characteristic intestinal histopathology called the attaching-and-effacing (A/E) lesion, which is characterized by microvilli effacement and intimate bacterial adherence to the epithelial membrane. The genes responsible for A/E lesion phenotype are encoded on 35.6-kb chromosomal pathogenicity island known as the locus of enterocyte effacement (LEE). The LEE island comprises approximately 40 genes and encodes the components of a type III secretion system, various effector molecules, and the intimin adhesin which is encoded by the *eae* gene [4, 5].

EPEC strains are classified into typical and atypical based on the presence of the large virulence EPEC adherence factor (EAF) plasmid [6]. The EAF plasmid encodes a type IV fimbria known as the bundle-forming pilus (BFP) that mediate localized adherence (LA) to epithelial cells [7–9]. A 14-gene operon is necessary for BFP production [10], with *bfpA* encoding the major structural subunit (bundlin). A second operon on the EAF plasmid is the plasmid-encoded regulator (Per), consisting of three genes (*perA*, *perB*, and *perC* genes) [11], which activates genes within the LEE and the *bfp* operon [12–14].

Additional criteria for classifying isolates as EPEC include the detection of specific serogroups [6]. Classic EPEC O serogroups include O55, O86, O111, O114, O119, O125, O126, O127, O128, and O142. By multilocus enzyme electrophoresis analysis of allelic differences among housekeeping genes, typical EPEC strains have been subtyped into two major lineages, previously designated EPEC1 and EPEC2 [15, 16]. The EPEC1 includes widespread serotypes such as O55:H6 and O119:H6, whereas EPEC2 consists of serotypes with more limited occurrence such as O111:H2 and O114:H2. Based on a whole-genome phylogeny and analysis of type III secretion system effectors, typical EPEC strains have been demonstrated to cluster in three main lineages, designated EPEC1, EPEC2, and EPEC4 [17]. According to the phylogenomic analyses by Hazen et al. [17] the term atypical EPEC refers to a group of phylogenetically diverse isolates than often are more similar to *E. coli* of other pathovars than EPEC. However, although the term atypical EPEC may be considered misleading, atypical strains are identified by the presence of *eae* and the absence of the EAF probe sequence as well as the Shiga toxin-encoded genes [6].

A 1-kb cryptic region of EAF plasmid has been widely used as a genetic probe for EPEC detection [18]. However, some EPEC strains, as demonstrated by BFP production, may in fact harbor an EAF plasmid lacking the EAF probe sequence [19, 20], which makes the differentiation between typical and atypical complex [21]. Other EPEC strains harbor an EAF plasmid that shares a conserved backbone, and is in many ways similar to pMAR7 plasmid of EPEC1 strain E2348/69 and pB171 plasmid of EPEC2 strain B171, but with inactivating deletions in the *bfp* and *per* operons that are required for LA [22, 23]. Such strains are phenotypically “atypical”, since they do not produce a typical LA pattern on epithelial cells, even though they carry probe-detectable EAF plasmids. In this study, we report the genetic analysis of the EAF plasmid-encoded genes in a collection of EPEC clinical isolates.

## Results and discussion

In this study, we analyzed a collection of 222 EPEC clinical isolates, including strains of the classic and nonclassic EPEC serotypes, which were previously classified as typical (*n* = 70) or atypical (*n* = 152) by EAF probe reactivity. Initially, all 222 strains were screened for the EAF probe sequence by using a PCR with primers located 100 nucleotides upstream (5′-CGCCATTTATTTTAAGACGAACA) and 82 nucleotides downstream (5′-CGCTTCTGCTTTTGACGG) the EAF sequence of pMAR2 [24]. As shown in Tables 1 and 2, the majority of typical strains yielded the expected 1260-bp amplicon. Interestingly no PCR product was obtained from a subset of 30 typical strains, 22 of which belonged to O119 serogroup. To verify this, a new primer located 20 nucleotides upstream *perB* (5′- GAGCACTCGAAATGAAGAAC) was designed to include the region between *perBC* and EAF sequence. All 30 typical strains showed a PCR product approximately 1.7-kb smaller than the expected (3.9 kb). DNA sequencing revealed the presence of a 1652 pb deletion not previously reported, in the region between 1093-bp downstream *perC* and 616-bp of the EAF fragment. As expected all the atypical strains were PCR-negative.

**Table 1:** Results of PCR and hybridization for EAF-encoded genes among typical EPEC strains

Clonal group	Serotype	No. of strains	Source	Presence or absence of:				IS element upstream of *per*	Presence or absence of:					*perBC-*EAF PCR product
				*bfpA* (probe)	*bfpA* (PCR)	*bfpG* (PCR)	*trcP* (probe)		*per* (probe)	*perA* (PCR)	EAF (PCR)	*orf35–36* (probe)	*orf61–62* (probe)	
EPEC1	O55:H6, NM	13	Patients	+	+	+	–	IS*1*(νξ)	+	+	+	+	+	3.9-kb
EPEC1	O55:NM	1	Control	+	+	+	–	IS*1*(νξ)	+	+	+	+	+	3.9-kb
EPEC1	O86:NM, H34	2	Patients	+	+	+	+	IS*1*(νξ)	+	+	+	+	+	3.9-kb
EPEC2	O111:NM	5	Patients	+	+	+	+	IS*1*(νξ)	+	+	+	+	+	3.9-kb
EPEC2	O111:H2	4	Patients	+	+	+	+	IS*1*(νξ)	+	+	+	+	+	3.9-kb
EPEC1	O119:H6, NM	16	Patients	+	+	+	–	IS*1*(νξ)	+	+	–	+	–	2.2-kb
EPEC1	O119:H6	1	Patient	–	+	+	–	IS*1*(νξ)	–	+	–	+	–	2.2-kb
EPEC1	O119:H6, NM	5	Patients	+	+	+	–	IS*1*(νξ)	+	+	–	–	+	2.2-kb
EPEC1	O119:H6	2	Controls	+	+	+	+	IS*1*(νξ)	+	+	+	+	–	3.9-kb
EPEC1	O119:H6	1	Control	+	+	+	+	IS*1*(νξ)	–	+	+	+	–	3.9-kb
EPEC1	O127:H6, NM	3	Controls	+	+	+	–	IS*1*(νξ)	+	+	+	+	–	3.9-kb
EPEC1	O127:NM	1	Patient	+	+	+	–	IS*1*(νξ)	+	+	+	+	–	3.9-kb
Unknown	O2:H2, H45	2	Patients	+	+	+	–	IS*1*(νξ)	+	+	+	+	–	3.9-kb
Unknown	O101:H33	1	Control	+	+	+	–	IS*1*(νξ)	+	+	+	+	–	3.9-kb
Unknown	O145:HNT	1	Control	+	+	+	–	IS*1*(νξ)	+	+	–	+	–	2.2-kb
Unknown	O162:NM, H33	2	Patients	+	+	+	–	IS*1*(νξ)	+	+	+	+	–	3.9-kb
Unknown	O157:HND	2	Patients	+	+	+	–	IS*1*(νξ)	+	+	–	+	–	2.2-kb
Unknown	ONT:H45; HND	3	Patients	+	+	+	–	IS*1*(νξ)	+	+	+	+	–	3.9-kb
Unknown	ONT:HND	5	Patients	+	+	+	–	IS*1*(νξ)	+	+	–	+	–	2.2-kb
EPEC1	E2348/69 (O127:H6)			+	+	+	–	IS*1*(νξ)	+	+	+	+	+	3.9-kb
EPEC2	B171 (O111:NM)			+	+	*+*	*+*	IS*1*(νξ)	+	+	+	*+*	*+*	3.9-kb

*NM* Nonmotile; *ND* Nondetermined; *NT* Nontypeable

**Table 2:** Characteristics of aEPEC strains positive for EAF plasmid-encoded genes either by PCR or hybridization assay

Strain	Serotype	Source	Presence or absence of:				IS element upstream of *per*	Presence or absence of:					HEp-2 adhesion pattern
			*bfpA* (probe)	*bfpA* (PCR)	*bfpA-IS66*	*trcP* (probe)		*per* (probe)	*perA* (PCR)	EAF (PCR)	*orf35–36* (probe)	*orf61–62* (probe)	
A103	O9:HND	Patient	–	–	–	+	*IS1294*	+	+	–	–	–	–
A148	O37:NM	Patient	–	–	–	–	*IS1294*	+	+	–	–	–	–
A141	O49:HND	Patient	–	–	–	–	*IS1294*	+	+	–	+	–	–
A69	O55:NM	Patient	+	+	–	+	*IS1294*	+	+	–	–	–	LAL
A152	O96:NM	Control	–	–	–	–	*IS1294*	+	+	–	–	–	–
A144	O98:HND	Patient	–	–	–	+	*IS1294*	+	+	–	+	–	–
A140	O108:HND	Control	+	–	+	–	*IS1294*	+	+	–	–	–	LAL
A60	O119:H2	Patient	+	–	+	*–*	*IS1294*	+	+	–	*–*	*–*	LAL
A62	O119:H2	Patient	+	–	+	*–*	*IS1294*	+	+	–	*–*	*–*	LAL
A66	O119:H2	Control	+	–	+	*–*	*IS1294*	+	+	–	*–*	*–*	LAL
A67	O119:H2	Patient	+	–	+	*–*	*IS1294*	+	+	–	*–*	*–*	LAL
A75	O119:HND	Patient	+	–	+	*–*	*IS1294*	+	+	–	*–*	*–*	LAL
A90	O119:HND	Patient	+	+	–	–	*IS1294*	+	+	–	–	–	LA
A111	O119:HND	Patient	+	–	+	*–*	*IS1294*	+	+	–	*–*	*–*	LAL
A127	O119:HND	Patient	+	–	+	*–*	*IS1294*	+	+	–	*–*	*–*	LAL
A131	O119:HND	Patient	+	–	+	*–*	*IS1294*	+	+	–	*–*	*–*	LAL
A97	O128:HND	Patient	–	–	–	–	*IS1294*	+	+	–	–	–	–
A126	O128:NM	Patient	–	–	–	–	*IS1294*	+	+	–	–	–	–
A11	O142:NM	Patient	+	+	–	+	*IS1294*	+	+	–	+	–	LA
A129	O142:HND	Patient	+	+	–	+	*IS1294*	+	+	–	+	–	LAL
A124	O157:NM	Patient	–	–	–	–	*IS1294*	+	+	–	–	–	–
A95	ONT:H21	Patient	+	+	–	+	*IS1294*	+	+	–	–	–	LAL
A65	ONT:NM	Control	+	+	–	–	*IS1294*	+	+	–	–	–	LAL
A74	ONT:NM	Patient	+	+	–	–	*IS1294*	+	+	–	–	–	LAL
A63	ONT:HND	Patient	+	+	–	–	*IS1294*	+	+	–	+	+	LAL
A64	ONT:HND	Patient	+	+	–	–	*IS1294*	+	+	–	+	+	LAL
A78	ONT:HND	Patient	+	+	–	–	*IS1294*	+	+	–	+	–	LAL
A146	ONT:HND	Control	+	+	–	–	*IS1294*	+	+	–	+	–	LAL
A57	ONT:HND	Patient	–	–	–	–	*IS1294*	+	+	–	–	–	–
A16	ONT:HND	Control	–	–	–	–	*IS1294*	+	+	–	+	–	–
A118	ONT:HND	Patient	–	–	–	–	*IS1294*	+	+	–	–	–	–
A136	ONT:HND	Patient	–	–	–	–	*IS1294*	+	+	–	+	–	LAL

*ND* Nondetermined; *NM* Nonmotile; *LA* Localized adherence patter; *LAL* LA-like pattern

**Fig. 1:**
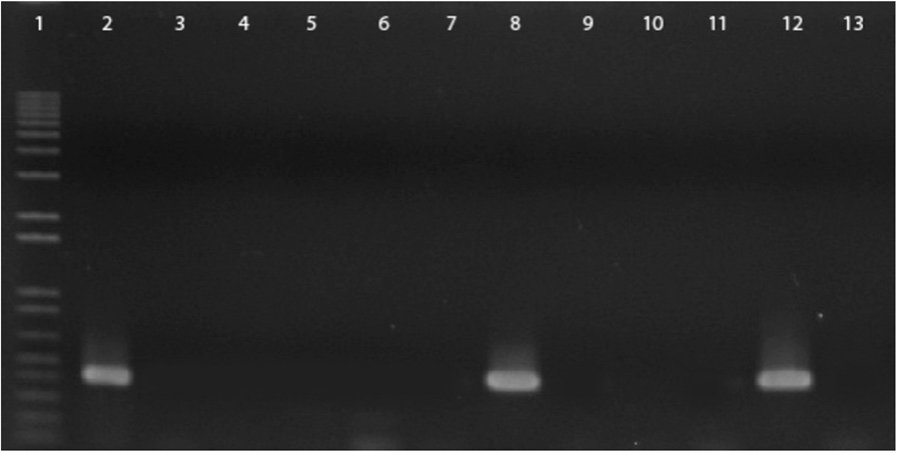
Agarose gel electrophoresis of the RT-PCR products of O119 and O142 atypical EPEC strains. Lane1, 1-kb ladder plus (Gibco, BRL); lane 2, E2348/69 strain (positive control); lane 3, A60 strain; lane 4, A62 strain; lane 5, A66 strain; lane 6, A67 strain; lane 7, A75 strain; lane 8, A90 strain; lane 9, A111 strain; lane 10, A127 strain; lane 11, A131; lane 12, A11 strain; and lane 13, A129 strain

As shown in Tables 1 and 2, the *bfpA, bfpG,* and *per* genes, detected by PCR and colony blot hybridization, were present in all typical strains. In addition, 32 (21 %) atypical strains presented the *per* genes*,* and 20 (13.2 %) also presented the *bfpA* gene. Among the 32 strains, 18 strains belonged to the serotypes O119:H2, O119:HND, O142:H2, and ONT:HND atypical strains. Although 20 of 32 strains hybridized with the *bfpA* probe, *bfpA* could not be amplified by PCR from eight O119 strains, suggesting that the 3′end of the gene was deleted and replaced with an IS*66*-like element as described by Bortolini et al. [22]. To verify this, we carried out PCR with primers targeting the 5′end of *bfpA* and the IS66-like element previously described [22]. All the eight O119 strains yielded the expected amplicon, and DNA sequencing confirmed the presence of a 1053-bp IS element inserted into *bfpA* at position 262 with significant similarity to IS*66* (97 %). We evaluated the level of expression of *bfpA* by RT-PCR in the 20 *bfpA*-positive strains. The RT-PCR results showed that the *bfpA* gene was transcribed only by the strains carrying the intact *bfpA* gene sequence (Fig. 1). Two of these strains, A90 (O119:HND) and A11 (O142:NM), present the LA pattern. Considering the BFP production as a truly phenotype marker of typical EPEC strains, these strains are in fact typical EPEC rather than atypical EPEC as originally classified by the EAF-probe reactivity.

**Fig. 2:**
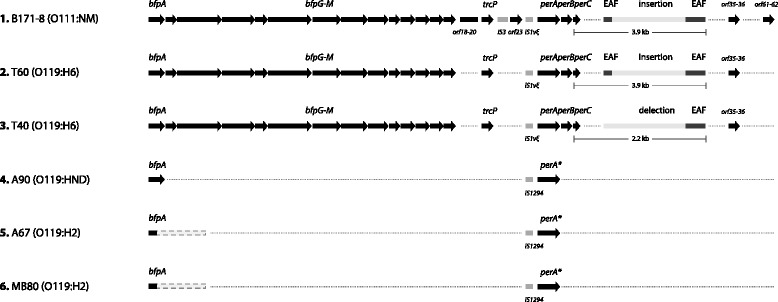
The *bfp*-*orf61-62* region of EAF plasmids from different EPEC strains. Diagram 1, EAF plasmid of B171-8 strain; diagrams 2 and 3, EAF plasmids of typical EPEC O119 strains (T60 and T40); diagrams 4 and 5, EAF plasmids of atypical EPEC O119 strains (A90 and A67); diagram 6, EAF plasmid of MB80 strain. Asterisks indicate truncated genes, and sequences for which no data are available are represented by dashed lines

The EAF plasmids from two well-studied EPEC strains have been sequenced [25, 26]. The major difference between pMAR7 (E2348/69, EPEC1 lineage) and pB171 (B171, EPEC2 lineage) is the presence of conjugative transfer (*tra*) genes on pMAR7, absent in pB171 [26]. Apart from the *tra* region, several other ORFs are present in pB171 but not in pMAR7, such as the region *orf35*–*36* (truncated homolog of the EHEC *toxB* gene), the region *orf61-62* (*gadB* [truncated] and *gadC* homologs), and the putative chaperonine *trcP* located between the *bfp* and *per* operons flanked by insertion (IS) elements. As shown in Tables 1 and 2, a subset of typical strains O55, O86, O111 and O119 strains were positive for one or more pB171 derived-probes, while most of atypical EPEC strains did not hybridize with one or more of the probes (Fig. 2).

The sequence of plasmid pB171 reveals that a partial IS*1*(νξ) homolog lies immediately upstream of the *per* operon [26]. This IS*1*(νξ) element is replaced by a partial IS*1294* in O119:H2 (MB80), O128:H2, and O142:H6 (O142#15) strains containing *perA* mutation frameshifts, leading to premature truncation and consequent inactivation of the gene [23]. Okeke et al. [23] developed a multiplex PCR which gives a 650-bp product in strains with the IS*1*(νξ) element and a 500-bp product in strains that have the IS*1294*, and a *Ssp*I-based PCR-RFLP typing which distinguishes normal *perA* alleles from those with O119:H2, O128:H2, or O142:H6 type-frameshift. As shown in Tables 1 and 2, all typical strains yielded a 650-bp product, while all 32 atypical strains with *perA* mutation frameshifts yielded a 500-bp product. In addition, all typical strains showed a *perA* RFLP pattern similar to that of control EPEC strain (E2348/69), while all 32 *perA* probe-positive atypical strains produced a pattern consistent with that of the MB80. The entire *perA* gene was amplified and sequenced in all 32 atypical strains, and DNA sequencing confirmed the presence of *perA* mutation identical to that of MB80 strain. These results suggest that the presence of an inactive *perA* gene could be used to differentiate typical from atypical strains. Interestingly, most strains carrying vestiges of EAF plasmid belong to the same serogroups as typical EPEC, suggesting that these strains may constitute a group of EPEC strains that carry a defective EAF plasmid rather than atypical EPEC isolates.

## Conclusion

Our data show that typical O119 strains, which were the most prevalent isolates in São Paulo, in the past [27], may contain a deletion within the EAF probe sequence not previously reported. This new finding suggests that care should be taken when using the previously described EAF PCR assay [28] in epidemiological studies for the detection of typical O119 strains. In addition, we were able to confirm that some atypical strains carry vestiges of the EAF plasmid [29–31].

## Methods

### Strains

The strains examined in this report were isolated during epidemiological studies of acute diarrhea in children <2 years of age conducted in different regions of Brazil in 1999–2009 [2, 32–34]. These strains were identified by colony hybridization with *eae* and/or EAF probe sequences and serotyped, and most of them had also been characterized by the presence of LEE-associated DNA sequences, and adherence to HEp-2 cells [34, 35]. Strains were grown overnight at 37 °C in 5 ml of Luria-Bertani broth with shaking. Genomic DNA was isolated using the HiYield Genomic DNA Mini kit (Real Biotech Corporation, Taiwan), and was used as the template for the PCR assays.

### Ethics statement

The study was approved by the ethics committee of the Universidade Federal de São Paulo, Brazil. Stool samples were obtained with the written informed consent from the parents or guardians of the children.

### Screening for EAF plasmid genes

The presence of EAF plasmid-encoded genes was determined by colony hybridization and PCR. Colony hybridizations were performed under high-stringency conditions at 65 °C employing probes that were labeled by random priming with [α−^32^P] dCTP as described previously [32]. Fragments probes for *bfpA* and *perABC*, were prepared from plasmid clones as described previously [22]. Probes for *bfpG*, *trcP*, *orf35–36*, and *orf61–62*, were prepared by PCR primers as described [23] using strain B171 as the template. The DNA fragments were purified, labeled with [α−^32^P] dCTP with a DNA labeling kit (Amersham Pharmacia Biotech Inc., EUA) and used as probes. Blots were hybridized in a solution containing the labeled probe (10^5^ cpm), 5 × standard saline citrate (SSC), 2 × Denhardt’s solution (Invitrogen), 0.1 % sodium dodecyl sulfate (SDS), and 5 mg/ml of salmon sperm DNA for 16 h at 65 °C. After hybridization, washes were done in aqueous solution with 2 × SSC with 0.1 % SDS and exposed to X-ray film.

The *bfpA* gene was amplified by PCR with primers bfpA_114F (GTCTGCGTCTGATTCCAATA) and bfpA_521R (TCAGCAGGAGTAATAGC) as previously described [36].

The entire *perA* gene was amplified by PCR with primers K1693 (CCCAAGCTTTGGCAATGTTCCTTGTGT) [23] and perA-24F (AACAAACGCGCATGAAGGTG) [22]. The 770-bp amplicon from *perA*-positive strains was digested with the restriction enzyme *Ssp*I, and restriction fragment length polymorphism (RFLP) analysis was performed by agarose gel electrophoresis.

The *per* upstream region to IS*1*(νξ) was amplified by PCR with primers K1547 (TGAGTCACCTCTGCCTGAG) and K1549 (TGGATTCTATTGTGTATTCGG), and the *per* upstream region to IS*1294* was amplified by PCR with primers K1978 (TGTGAGAGCTTCTCAGCA) and K1549 (TGGATTCTATTGTGTATTCGG) as previously described [23].

### RNA extraction and RT-PCR assays

Total RNA was extracted after bacterial growth in LB broth for 18 h at 37 °C with the RNase Mini extraction kit (Qiagen) according to the manufacturer’s instructions. After extraction, approximately 1 μg of total RNA was digested with DNase I (Qiagen) for 30 min at 37 °C, and the enzyme was then inactivated by adding 1 μl of 25 mM EDTA and heating the solution at 65 °C for 10 min. To obtain the cDNA, the SperScript III One Step RT-PCR System with Platinum *Taq* DNA polymerase (Invitrogen) was used according to the manufacturer’s specifications. Primers for 16S ribosomal protein were used to control PCR [37], and the assay was then carried out with primers bfpA_114F (GTCTGCGTCTGATTCCAATA) and bfpA_521R (TCAGCAGGAGTAATAGC) as previously described [36]. PCR products were analyzed by 1 % agarose gel electrophoresis.

### DNA sequencing

Nucleotide sequencing of the PCR products was performed at the Centro de Estudos do Genoma Humano-USP, São Paulo. Nucleotide sequence data were analyzed using SeqMan and MegAlign software and the BLAST tool (http://www.ncbi.nlm.nih.gov/BLAST).

### Nucleotide sequence and accession number

The DNA sequences for the EAF region of ypical EPEC O119 strains (T60 and T40) are availability in NCBI database under accession numbers KT595240 and KT819171.

## Abbreviations

EPEC: Enteropathogenic *Escherichia coli*
EAF: *E. coli* adherence factor plasmid
Bfp: Bundle-forming pilus
Per: Plasmid-encoded regulator
